# The Prevalence of Neonatal Near Misses in Rabat, Morocco

**DOI:** 10.7759/cureus.34486

**Published:** 2023-02-01

**Authors:** Kawtar Chafik, Fatima Aslaou, Fatima Barich, Fatim Zahra Laamiri, Ilham El Ouardighi, Amina Barkat

**Affiliations:** 1 Research Team on Health and Nutrition of Mother and Child, Faculty of Medicine and Pharmacy, Mohammed V University, Rabat, MAR; 2 Research, Higher Institute of Nursing Professions and Health Techniques, Rabat, MAR; 3 Laboratory of Health Sciences and Technology, Hassan First University of Settat, Higher Institute of Health Sciences of Settat, Settat, MAR

**Keywords:** morocco, newborn health, low birth weight, apgar score, neonatal near miss events

## Abstract

Background: The concept of near-miss neonatal (NMN) is a potentially useful approach in assessing the quality of newborn care. However, data collected on the status of NMN cases in Morocco is scarce.

Objective: The objective of this study is to determine the prevalence of NMN cases among live births at the University Hospital of Rabat, Morocco.

Materials and methods: An observational cross-sectional study was conducted on 2676 newborns born at the University Hospital of Rabat, Morocco, and admitted to the National Reference Center of Neonatology and Nutrition (NRCN) from January 1 to December 31, 2021. The main inclusion criteria were the pragmatic and/or management markers of the definition of NMN. Data were extracted using a structured, pre-tested checklist, entered into EpiData, and exported to Statistical Software for the Social Sciences (SPSS) version 23 (IBM Corp., Armonk, NY), and descriptive statistics were performed.

Results: Among the 2676 selected live births, 2367 were NMN cases (88.5%; 95% CI: 88.3-90.7). More than half of new mothers (57.5%) were referrals, 59.9% of women were multiparous, and 78.5% had under four prenatal care consultations. Obstetric problems affected 373 of the women during pregnancy. A pragmatic criterion was met in 43.6% of NMN situations. Among the management criteria, the use of intravenous antibiotics was the most common factor at 56.0%.

Conclusions: This study revealed a high prevalence of NMN. Therefore, concerted efforts are needed to improve maternal health care services, including early identification of complications and appropriate management.

## Introduction

The neonatal period is the most vulnerable time for infants. In 2019, 2.4 million children worldwide died during the first month after birth, approximately 6700 newborn deaths per day [1]. One million of these children die on the day of birth and nearly two million in the first week of life. The vast majority of these deaths occur in low-resource settings. In Sub-Saharan Africa, neonatal mortality accounts for 36% of all deaths of children under five years of age [1]. For every newborn who dies, up to eight others suffer life-threatening complications but survive (called "near-miss neonatal" [NMN]) [2,3]. A 2018 United Nations International Children's Emergency Fund (UNICEF) report established that the chances of newborn survival vary greatly depending on location [4]. To mitigate this, the Sustainable Development Goals (SDGs) call for an end to preventable newborn and under-five deaths and for all countries to have a neonatal mortality rate of 12 or fewer deaths per 1,000 live births by 2030 [1].

Morocco is among the countries that have prioritized maternal and neonatal morbidity and mortality issues. In 2018, a decrease in infant mortality has been achieved, reaching 18 deaths per 1000 live births with 75% of deaths occurring during the neonatal period (13.65 per 1000) [5]. Despite the decrease, the morbidity rate remains high. The number of NMN survivors is estimated to be anywhere from three to 10 times higher than the number of neonatal deaths [6]. Some researchers have defined NMN as a morbid event that nearly results in the death of a newborn during the neonatal period where he or she survives by chance or with quality care [6-8]. Causes may include illness, intervention, and organ dysfunction. The recommended pragmatic criteria for an NMN in newborns surviving four weeks after birth include a birth weight of less than 1750 g, gestational age of fewer than 33 weeks, or an Appearance, Pulse, Grimace, Activity, and Respiration (APGAR) score of less than 7 at five minutes. In addition to the pragmatic criteria, management markers include the use of intravenous therapeutic antibiotics, continuous nasal positive airway pressure, intubation, phototherapy within the first 24 hours, cardiopulmonary resuscitation, vasoactive drugs, anticonvulsants, surfactant administration, blood products, and steroids to treat refractory hypoglycemia [9,10]. The pragmatic criteria and management markers were developed by Pileggi-Castro et al. and were found to have a sensitivity of 93% and specificity of 97% [9].

Until now, very little research has been done on NMN, and data on NMN cases are scarce in Morocco as well as low- and middle-income countries. Considering its usefulness as a tool for improving the quality of neonatal care, the objective of this study is to determine the prevalence of NMN cases among live births born at the level of the University Hospital Ibn Sina of Rabat, Morocco, in 2021. Providing data on the current situation of neonatal near-miss cases in Morocco would help in the development of policies that could contribute to the reduction of neonatal mortality in the country and the achievement of the SDGs.

## Materials and methods

Study design and setting

An observational cross-sectional study was conducted on 2676 newborns born at the University Hospital of Rabat, Morocco, and admitted to the National Reference Center of Neonatology and Nutrition (NRCN), Department of Medicine and Neonatal Resuscitation of the Children's Hospital of Rabat, Morocco, from January 1 to December 31, 2021.

Study population

Of the 2676 newborns admitted to the NRCN, Department of Medicine and Neonatal Resuscitation of the Children's Hospital of Rabat, Morocco, during the study period, 2367 newborns and their mothers were selected and examined, an exhaustive sampling.

Data collection

The study included patients admitted to the Medicine and Neonatal Resuscitation of the Children’s Hospital of Rabat, Morocco, who presented at least one pragmatic and/or management criteria of the definition of NMN. They were followed for up to 28 days to determine the chances of survival. The study used both pragmatic and management criteria for defining NMN [10]. These criteria include pragmatic criteria (birth weight less than 1750 g, gestational age less than 33 weeks, or APGAR score less than 7 at five minutes) and/or management criteria (the use of intravenous therapeutic antibiotics, continuous nasal positive airway pressure, intubation, phototherapy within the first 24 hours, cardiopulmonary resuscitation, vasoactive drugs, anticonvulsants, administration of surfactants, blood products, steroids to treat refractory hypoglycemia, or any surgical intervention) [10].

The data were extracted using a pre-tested structured questionnaire. Variables that may contribute to the level of risk for NMN include maternal age, socioeconomic status, level of education, occupation, social security coverage, place of residence, reference, marital status, parity, antenatal care visit (ANC), history of abortion, previous stillbirth, premature rupture of the membrane (PROM), gestational age (GA), multiple deliveries, pathologies during pregnancy (hypertension, diabetes, anemia, and asthma), mode of delivery presentation, birth weight, APGAR score at birth and 5 minutes after birth, use of intravenous therapeutic antibiotics, continuous nasal positive airway pressure, intubation, phototherapy within the first 24 hours, cardiopulmonary resuscitation, vasoactive drugs, anticonvulsants, administration of surfactants, blood products, steroids to treat refractory hypoglycemia, and any surgical intervention.

Statistical analysis

Prior to analysis, double-check data entry was conducted to increase the validity and accuracy of data entry. The data was then entered into EpiData and exported to Statistical Software for the Social Sciences (SPSS version 23.0, IBM Corp., Armonk, NY), and descriptive and analytical procedures were performed, followed by descriptive statistics such as frequencies and cross-tabulations. Data analysis was performed using the chi-square test and Fisher's exact test where appropriate, with a significance level of 5%. Results were presented in text and table format.

Ethical considerations

The study protocol was approved by the Ethics Committee of the Faculty of Medicine and Pharmacy at Mohammed University of Rabat, Morocco (Ethics approval number: C64/20). Data collectors and supervisors were trained, and a pretest was conducted. About 20 participants were informed of the study objectives and methods, and oral and written consent was obtained. Confidentiality of their participation and anonymity of their data were assured. Throughout the data collection process, data collectors were supervised, and regular meetings were held to address any issues.

## Results

Among the 2676 newborns admitted to the NRCN, Department of Medicine and Neonatal Resuscitation of the Children's Hospital of Rabat, Morocco, during the study period, 2367 (88.5%) (95% CI: 88.3%-90.7%) were classified as NMN cases, 133 (5%) were classified as non-NMN, and 176 (6.5%) died (Table 1).

**Table 1:** Prevalence of NMN in NRCN, Rabat, Morocco, in 2021 (n = 2676) NRCN: National Reference Center of Neonatology and Nutrition; NMN: Near-miss neonatal.

	Number	Percent (%)
Total admitted in NRCN	2676	100
Total near-miss events in newborns admitted to NRCN during the study period	2367	88.5
Total number of deaths during the study period	176	6.5
Total number of cases classified as not NMN	133	5

Sociodemographic characteristics

More than half of the mothers of newborns were referrals (57.5%; 95% CI: 55.7-99.6). Almost 70% were between 20 and 34 years of age (69.1%; 95% CI: 67.3-71) and just over 60% had a primary education level or less (62%; 95% CI: 98.9-59.6). The distribution by place of residence was dominated by urban areas (67.7%; 95% CI: 65.8-69.6). In addition, more than three-quarters of the mothers had a low socioeconomic status (76%; 95% CI: 98.9-99.6), and more than a third did not benefit from medical coverage (38.1%; 95% CI: 36.3-40) (Table 2).

**Table 2:** Sociodemographic characteristics among mothers’ population Note: Values are expressed as numbers and percentages.

Variable Categories	Neonatal Near Miss N (%) (N = 2367)	95% Confidence Interval (CI)
Age in years (n = 2367)		
Less than 20	113 (4.9)	3.9–5.7
Between 20 and 34 years	1636 (69.1)	67.3–71
≥35	618 (26.1)	24.4–27.8
Residence (n = 2367)		
Urban	1603 (67.7)	65.8–69.6
Rural	700 (29.6)	27.7–31.3
Suburban	64 (2.7)	2.0–3.4
Maternal education level (n = 2367)		
None	630 (26.6)	24.9–28.4
Primary	838 (35.4)	33.5–37.3
Secondary	724 (30.6)	28.8–32.4
Higher	175 (7.4)	6.4–8.4
Socioeconomic status (n = 2367)		
Low	1799 (76.0)	74.3–77.7
Medium	562 (23.7)	22.1–25.4
High	6 (0.3)	0.1–0.5
Referral (n = 2367)		
No	1005 (42.5)	40.4–44.3
Yes	1362 (57.5)	55.7–59.6
Marital status (n = 2367)		
Married	2349 (99.2)	98.9–99.6
Single	18 (0.8)	0.4–1.1
Coverage (n = 2678)		
No	902 (38.1)	36.3–40.0
Medical assistance regime	935 (39.5)	37.4–41.4
National Fund of Social Welfare Organizations	364 (15.4)	13.9–16.8
National Social Security Fund	151 (6.4)	5.3–7.4
Private insurance	13 (0.5)	0.3–0.9
Other	2 (0.1)	0.0–0.2
Mother’s occupation (n = 2367)		
No	2287 (96.6)	95.8– 97.3
Yes	80 (3.4)	2.7–4.2
Father’s occupation (n = 2367)		
No	59 (2.5)	1.9–3.1
Yes	2582 (96.4)	96.7–98.0

Obstetrical/gynecological and medical characteristics

About 59.9% of the mothers participating in the study were multiparous, 2144 (90.6%) had at least one ANC consultation, of which more than three-thirds (1859, 78.5%) had incomplete (<4) ANC consultations. About 618 (26.1%) of the mothers had pathologies during the index pregnancy namely hypertension (49%), diabetes (47.4%), complications leading to cesarean delivery (40.4%), and PROM (35.5%) (Table 3).

**Table 3:** Obstetric characteristics of mothers ANC: Antenatal care.

Variable Categories (n = 2367)	Frequency	Percent (%)
Parity		
Primiparous	949	40.1
Multiparous	1419	59.9
Antenatal care follow-up		
Yes	2144	90.6
No	223	9.4
Frequency of ANC follow-up		
<4	1859	78.5
≥4	508	21.5
History of abortion (n = 2367)		
Yes	442	18.7
No	1925	81.3
History of neonatal death (n = 2367)		
Yes	20	0.8
No	2347	99.2
History of stillbirth (n = 2367)		
Yes	67	2.8
No	2300	97.2
Pathologies during pregnancy (n = 618)		
Hypertension	302	49
Diabetes	293	47.5
Asthma	10	1.6
Anemia	13	2.1
Premature rupture of membrane		
No	1527	64.5
Yes	840	35.5
Presentation		
Vertex	2283	96.6
Non-vertex	84	3.4
Mode of delivery		
Vaginal deliveries	1411	59.6
Cesarean section	954	40.4

The magnitude of NMN cases

Characteristics of NMN Cases

Of the 2367 NMN events selected, 417 (17.6%) neonates had a birth weight of less than 1750 grams, 529 (22.3%) neonates had an APGAR score of less than 7 at 5 minutes, and 56 (2.4%) neonates had a GA of less than 33 weeks at birth. Among the management markers used, the use of intravenous therapeutic antibiotics was the most common marker at 1326 cases (56.0%), followed by the use of anticonvulsants at 1191 cases (50.4%) and cardiopulmonary resuscitation (CPR) at 937 cases (39.6%) (Table 4).

**Table 4:** Distribution of newborns admitted to the National Reference Center of Neonatology, Rabat, Morocco, in 2021 (n = 2367) APGAR: Appearance, Pulse, Grimace, Activity, and Respiration.

Criteria	Neonatal Near-Miss Cases	
	Frequency	Percent (%)
Pragmatic criteria		
APGAR score less than 7 at 5 minutes	529	22.3
Birth weight less than 1750 g	417	17.6
Gestational age less than 33 weeks	56	2.4
Management criteria		
Use of intravenous antibiotics up to 7 days and before 28 days of life	1326	56.0
Use of anticonvulsant	1191	50.4
Cardiopulmonary resuscitation	937	39.6
Nasal continuous positive airway pressure (NCPAP)	270	11.4
Use of phototherapy in the first 24 hours	188	8.0
Any intubation	174	7.3
Transfusion of blood derivatives	107	4.6
Surfactant	56	2.4
Use of corticosteroid for the treatment of refractory hypoglycemia	20	0.8
Any surgical procedure	12	0.5
Vasoactive	2	0.1

In addition, it was observed that only 26 cases had a combination of the three pragmatic criteria (Table 5).

**Table 5:** Association of near-miss pragmatic criteria in the study group APGAR: Appearance, Pulse, Grimace, Activity, and Respiration.

Combination of Pragmatic Criteria	No. of Neonates
All three	26
Birth weight less than 1750 g and gestational age less than 33 weeks	52
Birth weight less than 1750 g and APGAR score less than 7 at 5 minutes	47
Gestational age less than 33 weeks and APGAR score less than 7 at 5 minutes	29

## Discussion

This study highlighted the magnitude of NMN cases among live births born at the Ibn Sina University Hospital (Chis) of Rabat, Morocco, and admitted to the same center's National Reference Center of Neonatology and Nutritional Service of Medicine and Neonatal Resuscitation in 2021.

In our study, of 2676 neonates with complications admitted to the intensive care unit during the study period, 88.5% were NMN in which 17.6% had a birth weight of less than 1750 grams, 22.3% neonates had an APGAR score of less than 7 at 5 minutes, and 2.4% had a GA of less than 33 weeks at birth. Among the management criteria, the highest risk factor for NMN was the use of intravenous antibiotics at a risk increase of 56.0%, followed by the use of anticonvulsants at 50.4%, and cardiopulmonary resuscitation (CPR) at 39.6%. These observed high frequencies could be due to the study being conducted in a referral hospital center whose main recruitment comes from two maternity wards of Chis that receive women with high-risk pregnancies and a high flow of referrals.

The present study revealed the percentage of NMN cases to be 88.5%, which is 13 times higher than the percentage of neonatal mortality found in the same study site (6.5%). This result is consistent with studies conducted in India (87.6%) and Ghana (86.5%) [11,12]. The agreement between these studies could be due to the fact that they were conducted in neonatal intensive care units or referral hospitals that manage high-risk women. However, the proportion of NMN found in the present study is higher than that reported in other studies (22% in Northeast Brazil, 26.7% in Southwest Ethiopia, and 1.72% in Australia) [12-14]. This inconsistency in the percentage of NMN could be attributed to the difference in the study settings. Most of the aforementioned studies were conducted in study settings with high-quality maternal and newborn health care (technologies and early detection of problems) where neonatal morbidities (NMN cases) tend to be low. The discrepancy may also be attributed to both the criteria used to identify NMNs and the period of observation.

In addition, these dissimilarities could be due to differences in the socioeconomic characteristics of the study population. The results of the study showed that more than half of the mothers of NMN cases were between 20 and 34 years of age (69.1%), and 26.1% were less than 35 years of age, which differs from the study conducted in Australia which states that women older than 35 years of age have more than a fourfold risk of having a case of NMN (adjusted odds ratio [AOR] = 4.03; 95% CI: 1.58, 10.31). Secondary analysis of the WHO Multicountry Survey on Maternal and Newborn Health showed that advanced maternal age significantly increased the risk of perinatal death [15].

It was also found that 57.5% of the mothers were referred from other facilities, which is consistent with the Abebe study in Ethiopia which found that women referred from other facilities had more than seven times the risk of having a case of NMN (AOR = 7.53; 95%CI: 3.99-14.22) [16]. This can also be explained by the particularity of our working site since the study was conducted in a national reference center. It should also be noted that the socioeconomic context is a determining factor in the health of the mother-newborn couple; our results revealed that 62% of the mothers participating in the study have a primary level of education and less, 76% had a low socioeconomic level, and 38.1% were without medical coverage. An association between maternal education and neonatal mortality, particularly in low-income countries, has been documented in other studies [17,18]. In addition, educated mothers are more likely to have a higher socioeconomic status, have better knowledge of healthy behaviors, and use health care appropriately [19].

In this study, it was found that more than half (59.9%) of the mothers participating in the study were multiparous, in contrast to the results of the study conducted in northern Ethiopia, which showed that newborns born to primiparous women were 2.55 times more likely to suffer from NMN than their counterparts [16]. However, a recent prospective cohort study in Ethiopia reported that high multiparity was a risk factor for perinatal mortality in women with NMN [20]. Other research has shown that multiparous mothers have a high risk of developing complications during delivery, which exposes newborns to adverse outcomes [21,22].

In relation to pregnancy follow-up, the results showed that more than three-fourths of mothers of NMN cases (78.5%) had less than four ANC visits, which corroborates with the results of studies conducted in northern Ethiopia and Brazil demonstrating that incomplete antenatal visit attendance is a risk factor for having a case of NMN [12,23]. Results from a prospective analytical cohort study conducted in a maternity hospital for high-risk pregnancies in northeastern Brazil showed that attendance at fewer than six ANC visits was statistically significantly associated with NMN, increasing the risk of NMN by fourfold [12]. Attendance at four or more ANC visits was protective, whereas inadequate ANC visits increased neonatal mortality and adverse birth outcomes [24].

The study also showed that almost half of the mothers of NMN cases presented with pathologies during pregnancy such as hypertension, diabetes, or obstetrical complications leading to cesarean delivery and PROM. These results are consistent with the findings of studies from Brazil, Ethiopia, Uganda, and Australia [12-14,16,22]. This could be due to the fact that these complications during pregnancy can lead to fetal complications during intrauterine life, such as intrauterine growth retardation and preterm delivery, which increases the newborn’s risk of being underweight and causes birth asphyxia. In 2015, a systematic review and meta-analysis have shown that maternal and perinatal outcomes are often related [25]. Mothers at high risk of maternal complications often delivered by cesarean section and had a newborn with a low five-minute APGAR score. They were more likely to be admitted to a neonatal intensive care unit than women who had a cesarean delivery during the first stage of labor [25].

The study sample is not representative of the population of pregnant women in Rabat because the NNM was evaluated in a referral hospital for women with high-risk pregnancies. As a result, there are some limitations to the study. Additionally, the data corresponding to the socioeconomic variables and the frequency of prenatal visits were included and could be subject to recall bias.

## Conclusions

In this study, a higher prevalence of NNM cases was obtained at the NRCN in Rabat, Morocco. The data analyzed here can provide information that can contribute to the global and local neonatal and maternal morbidity research agenda about the most frequent complications related to NMN. This would allow for the institution of policies that could contribute to the reduction of NMN events and neonatal mortality. To increase the usefulness and applicability of the NMN concept, it is necessary to standardize its definition and identification criteria.
